# Artemisinin-based combination therapy in pregnant women in Zambia: efficacy, safety and risk of recurrent malaria

**DOI:** 10.1186/s12936-017-1851-7

**Published:** 2017-05-16

**Authors:** Michael Nambozi, Jean-Bertin Bukasa Kabuya, Sebastian Hachizovu, David Mwakazanga, Joyce Mulenga, Webster Kasongo, Jozefien Buyze, Modest Mulenga, Jean-Pierre Van Geertruyden, Umberto D’Alessandro

**Affiliations:** 1 Department of Clinical Sciences, Tropical Diseases Research Centre, P.O Box 71769, Ndola, Zambia; 2 Global Health Institute, University of Antwerp, Antwerp, Belgium; 3 Institute of Tropical Medicine, Antwerp, Belgium; 4 Medical Research Council Unit, Serekunda, Gambia; 5 London School of Hygiene and Tropical Medicine, London, UK

**Keywords:** Zambia, Sub-Saharan, Africa, Artemisinin-combination therapy, Treatment failure

## Abstract

**Background:**

In Zambia, malaria is one of the leading causes of morbidity and mortality, especially among under five children and pregnant women. For the latter, the World Health Organization recommends the use of artemisinin-based combination therapy (ACT) in the second and third trimester of pregnancy. In a context of limited information on ACT, the safety and efficacy of three combinations, namely artemether–lumefantrine (AL), mefloquine–artesunate (MQAS) and dihydroartemisinin–piperaquine (DHAPQ) were assessed in pregnant women with malaria.

**Methods:**

The trial was carried out between July 2010 and August 2013 in Nchelenge district, Luapula Province, an area of high transmission, as part of a multi-centre trial. Women in the second or third trimester of pregnancy and with malaria were recruited and randomized to one of the three study arms. Women were actively followed up for 63 days, and then at delivery and 1 year post-delivery.

**Results:**

Nine hundred pregnant women were included, 300 per arm. PCR-adjusted treatment failure was 4.7% (12/258) (95% CI 2.7–8.0) for AL, 1.3% (3/235) (95% CI 0.4–3.7) for MQAS and 0.8% (2/236) (95% CI 0.2–3.0) for DHAPQ, with significant risk difference between AL and DHAPQ (p = 0.01) and between AL and MQAS (p = 0.03) treatments. Re-infections during follow up were more frequent in the AL (HR: 4.71; 95% CI 3.10–7.2; p < 0.01) and MQAS (HR: 1.59; 95% CI 1.02–2.46; p = 0.04) arms compared to the DHAPQ arm. PCR-adjusted treatment failure was significantly associated with women under 20 years [Hazard Ratio (HR) 5.35 (95% CI 1.07–26.73; p = 0.04)] and higher malaria parasite density [3.23 (95% CI 1.03–10.10; p = 0.04)], and still women under 20 years [1.78, (95% CI 1.26–2.52; p < 0.01)] had a significantly higher risk of re-infection. The three treatments were generally well tolerated. Dizziness, nausea, vomiting, headache and asthenia as adverse events (AEs) were more common in MQAS than in AL or DHAPQ (p < 0.001). Birth outcomes were not significantly different between treatment arms.

**Conclusion:**

As new infections can be prevented by a long acting partner drug to the artemisinins, DHAPQ should be preferred in places as Nchelenge district where transmission is intense while in areas of low transmission intensity AL or MQAS may be used.

## Background

Malaria is a poverty-related disease and a major public health problem in many sub-Saharan African countries where over 90% of the cases worldwide are found. Pregnant women and children are at higher risk of malaria infection and of developing serious complications related to the disease. Malaria in pregnancy is associated with higher risk of maternal anaemia, low birth weight, spontaneous abortion, stillbirths and maternal mortality [1–3].

There are few treatments with known safety and efficacy for the treatment of malaria in pregnancy. Some anti-malarials known to be efficacious e.g. quinine, are not well tolerated, resulting in poor compliance and higher risk of treatment failures [4]. For other treatments, there are insufficient data as pregnant women are systematically excluded from treatment efficacy studies. Therefore, pregnant women lack proven effective and safe anti-malarial therapies [5]. In such context of limited information, and weighing risks and benefits, the World Health Organization (WHO) allows the use of artemisinin-based combination therapy (ACT) during the second and third trimester of pregnancy [1].

To confirm this expert opinion, we assessed the safety and efficacy of three artemisinin-based combinations, namely mefloquine–artesunate (MQAS), dihydroartemisinin–piperaquine (DHA–PQ) and artemether–lumefantrine (AL), in pregnant women in the second or third trimester with a confirmed *Plasmodium falciparum* malaria infection. This study was part of a multi-centre trial carried out also in Burkina Faso, Ghana and Malawi. Each site tested 3 ACT medicines so that each country dataset could be analysed separately [6] and give detailed site specific data. This paper reports results collected in the Zambian site, Nchelenge, Luapula Province. Knowing that anti-malarial drug’s efficacy depends not only on the parasite susceptibility to the drug and on its blood concentration but also on the host’s immunity which may be affected by factors such as pregnancy itself, age, parasite density and malaria transmission intensity, the impact of these factors on the treatment outcome were assessed [7, 8]. The results of this study provide the national policy makers the information for a wider and alternative choice of treatments to be used during pregnancy.

## Methods

The trial was conducted between June 2010 and August 2013 in Nchelenge district in Luapula Province, Zambia; one of the provinces where malaria prevalence is higher than the national average (32.1% vs 14.9% in 2012) [9]. Nchelenge district is located in the northern part of the province on the swampy shores of Lake Mweru, borders with the Democratic Republic of Congo (DRC) and has an estimated population of 178,000 inhabitants, mostly peasant farmers and/or fishermen. The district has three seasons: cool dry winter, hot dry and rainy season. Malaria transmission is perennial because of the presence of *Anopheles funestus* during the dry season and *Anopheles gambiae* in the wet season [10]. In 2012-2013, the entomological inoculation rate (EIR) was estimated at 70 infective bites/person/year [11], and annual malaria incidence at more than 700/1000 person years in the general population and more than 1900/1000 person years among under-five children [12]. The study protocol of this trial is been described in detail elsewhere [13]. Briefly, pregnant women aged at least 15 years, in the second and third trimester, with Hb ≥7 g/dL, HIV negative, a *P. falciparum* mono-infection of any density and irrespective of having symptoms (excluding illness at time of screening that required hospitalization such as severe malaria) were recruited into the trial and randomized to one of the following treatments: artemether–lumefantrine (AL), mefloquine–artesunate (MQAS) and dihydroartemisinin–piperaquine (DHA–PQ) using a randomization list provided of 300 participants in each arm. Sealed envelopes labeled with patient’s unique code and containing treatment allocation were provided according to randomization list. A woman was defined as symptomatic if any of the following were present: fever (temperature >37.5 °C) at baseline with parasitaemia (any density); parasite count >2000/µL, regardless of symptoms; at least 3 or more of the following symptoms: fever in the past 24 h, weakness/fatigue; muscle and/or joint aches, headache, convulsion, with parasitaemia of any density). Gestational age was estimated by symphysio-fundal height and then confirmed by obstetric ultrasound, including the fetal viability assessment [14, 15]. A blood sample of about 5 mL was collected before treatment for the assessment of haematological and biochemistry parameters. All study drugs were given on days 0, 1 and 2 under direct observation and according to the manufacturer’s recommendations (Eurartesim^®^ from Sigma-Tau Industrie Farmaceutiche Riunite S.p.A., 40 mg of dihydroartemisinin and 320 mg of piperaquine phosphate per tablet, 3 tablets once per day over 3 days; mefloquine–artesunate from Far-Manguinhos Ministério da Saúde-Fundação Oswaldo Cruzm, 100 mg artesunate and 220 mg mefloquine per tablet at 3 tablets once per day over 3 days; Coartem^®^ from Novartis Pharma AG, 20 mg artemether and 120 mg lumefantrine per tablet at 4 tablets twice per day over 3 days). After completing the 3-day treatment, patients were asked to return to the clinic for follow up visits on day 3, 7 and then once every week until day 63. At each visit, a medical history, and current clinical signs and symptoms were collected, including information on any adverse events (AE), a blood sample for malaria smears and dried blood spots (DBS) for later genotyping, for full blood counts (days 7, 14, 28 and 63 only) and for total bilirubin, alanine aminotransferase (ALAT) and creatinine (days 7 and 14 only). Rescue treatment (Quinine) for recurrent infections was according to local national guidelines [16]. (In Zambia, AL is used for treatment of uncomplicated malaria in second and third trimester of pregnancy). At the end of the active follow-up period, women were asked to continue with the antenatal clinic monthly or when they felt unwell until delivery. Recurrent malaria episodes after day 63 were treated with quinine.

Giemsa-stained thick and thin blood films were read independently by two readers, followed by a third reader in case of significant discrepancy. Parasite density was estimated by counting the number of asexual parasites per 200 white blood cells (WBCs) assuming a WBC count of 8000/µL. Total bilirubin, ALAT and creatinine were measured using Flexor Junior biochemistry analyzer. Full blood count was obtained using the Sysmex XT-2000i haematology analyzer. Haemoglobin (Hb) was measured using Hemocue (Angelholm, Sweden). For polymerase chain reaction (PCR) analysis, DBS were prepared on filter paper (Whatman 3MM), and were subsequently transported to the Institute of Tropical Medicine (ITM), Antwerp, Belgium, where centralized genotyping (GluRP, MSP2 and MSP1) was conducted [17]. Samples that failed to produce a result were classified as indeterminate.

Consent was obtained in all cases from study participants and/or legal representative for those between 15 and 17 years old. The study was approved by the Institutional Review Board of the ITM and the Ethics Committee of the Antwerp University Hospital. In addition, the study was also approved by Tropical Diseases Research Centre (TDRC) Ethics Review Committee, the Zambia Medicines Regulatory Authority and Zambia Ministry of Health. The trial was registered at clinicaltrials.gov (NCT00852423).

The primary endpoints were the PCR-adjusted cure rates at day 63 and the safety outcomes as described elsewhere [13]. AEs and serious AEs (SAEs) were recorded and monitored regularly throughout the study by an independent Data and Safety Monitoring Board (DSMB). Secondary endpoints were PCR-unadjusted cure rates at day 63, PCR adjusted and unadjusted time to treatment failure, asexual parasite clearance [18], gametocytaemia (prevalence and density) and Hb changes during follow up.

The study was designed to show that all 3 treatments had similar (PCR-adjusted) cure rates (within 5% difference), with 95% power for each of the 3 pair-wise comparisons and 80% power for the combined hypothesis that all treatments were therapeutically equivalent [13].

Data were captured into an electronic clinical record form (e-CRF) developed in MACRO (InferMed©). A statistical analysis plan was pre-specified before the database lock. For the primary outcome, three analysis populations were used: (1) per-protocol (PP), (2) intention-to-treat (ITT) that excluded lost to follow-up (LTFU)/withdrawals and missing/indeterminate PCR results, and (3) ITT with multiple imputations of LTFU/withdrawals and missing/indeterminate PCR results. The PP analysis was considered as the primary analysis approach. Major protocol violators, defined prior to analysis, were excluded from the PP analysis.

PCR-adjusted treatment failure rate between pair-wise treatment groups was compared using a Chi square test. The 95% exact confidence intervals for the difference in failure rates were determined. If the difference in true (PCR adjusted) failure rates was less than 5%, treatments were considered therapeutically equivalent. Briefly, risk difference was computed for the following groups: AL and DHAPQ; AL and MQAS; and MQAS and DHAPQ. The 95% confidence interval for a proportion was calculated using the Wilson score method. Baseline variables to be included in the Cox-regression model to compute the adjusted hazards of re-infection (new infection) and recrudescence were selected using the log-rank test for equality across strata. The covariates were included if the p value was 0.25 or less except study treatment dosage. The starting covariates were treatment, symptomatic malaria, parasite density, maternal age, gravidity, anaemia, study treatment dosage, gestational age, haematological and biochemical parameters. Covariates in the multivariable model that were not statistically significant (>0.05) were dropped off except where literature shows them as important variables [gravidity and gestational age (dropped for new infection)] to have in the final model. The proportion hazard assumptions for the Cox-regression model were evaluated using graphical approach [19].

The hematological and biochemistry profiles by day of follow-up were assessed using box-plots plotted at each time point. Differences in these parameters between treatment arms at each day of follow-up were assessed using Kruskal–Wallis test.

Firth logistic regression was used to assess impact of placental malaria (categorized as placental malaria or no infection) on birth outcomes (still birth, miscarriage, premature live delivery, intrauterine fetal death and term live birth) for separation and ‘empty cells’ in the model. A “stillbirth” was defined as a baby born dead after 24 weeks gestation; a baby born dead before 24 weeks gestation or during the 24th week was considered a “miscarriage”. “Preterm live born” was defined as a delivery before 37 weeks of gestation following echography. This was calculated as date of delivery minus date of echography (in weeks) plus gestational age determined through echography. Or based on the Ballard score which determines gestational age based on the sum of neuromuscular and physical scores [20]. A neonate with a score of 30 or lower was labeled “preterm” using this method. Logistic regression was used to assess impact of placental malaria (categorized as placental malaria or no infection) on birth weight. It was also used to assess risk factors for malaria. Placental malaria was classified as acute infection; chronic infection; past infection or no infection and analysed as binary outcome, placental malaria or no infection. For safety, all individuals having received at least one treatment dose were included and analysed in terms of proportions with Chi square test for the difference. Delivery related AEs, caesarean sections or reasons for caesarean sections and pregnancy outcomes were not included in the AE report. Also SAEs which were pregnancy related were excluded.

## Results

**Fig. 1:**
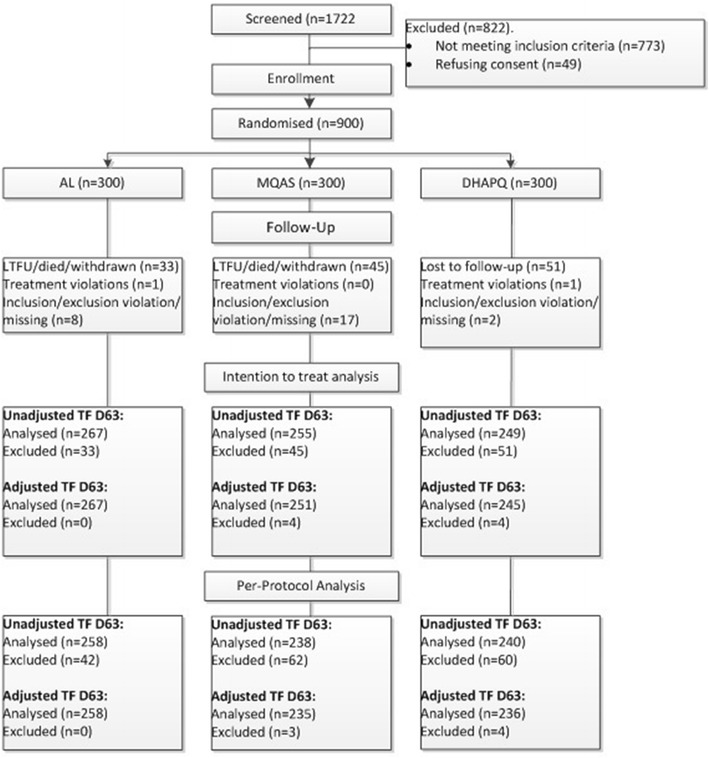
Trial flow chart of the PREGACT trial at Nchelenge, Zambia (2010–2014)

A total of 1722 pregnant women were screened for malaria infection, regardless of symptoms. Out of these, 900 met the inclusion criteria and were randomized to one of the three study arms: 300 to AL, 300 to MQAS and 300 to DHAPQ. The ITT analysis included 900 pregnant women. The PP analysis included 729 women, i.e. 258 in the AL, 235 in the MQAS and 236 in DHAPQ arms (Fig. 1). The main reasons for exclusion from the PP analysis were lost to follow-up and withdrawals. The baseline characteristics (age, gravidity, parasite density, Hb, symptoms) of the excluded patients were similar to those included in the PP analysis.

Baseline characteristics were similar between treatment arms (Table 1); all gravidities were equally represented and the median gestational age by obstetric ultrasound (echography) was 25.0 weeks [IQR: 20.5–29.0] and similar between the treatment arms. The median parasite density was 1540/µL (IQR: 480–4540) and similar between the treatment arms [AL 1360/µL (IQR: 480–4280), MQAS 1610/µL (IQR: 540–4340) and DHAPQ 1640/µL (IQR: 520–4880)]; over 40% of women had a parasite density ≥2000/μL and 49% were symptomatic.

**Table 1:** Baseline characteristics of pregnant women with malaria episode Nchelenge, Zambia (2010–2014)

	AL (N = 300)	MQAS (N = 300)	DHAPQ (N = 300)
Age (years): median (IQR)	20 (18–24)	19 (18–24)	20 (18–24)
Symptomatic malaria (%)	46.0	48.7	52.7
Fever (temperature ≥ 37.5 °C) (%)	2.0	3.3	3.7
Parasite density >2000/µL (%)	41.0	42.7	45.7
At least 3 symptoms^a^ (%)	11.0	12.3	12.0
Gametocytes present (%)	2.3	0	1.3
Parasite density (/µL): median (IQR)	1360 (480–4280)	1610 (540–4340)	1640 (520–4880)
Haemoglobin (g/dL): median (IQR)	10.0 (9.1–11.0)	10.0 (9.0–10.9)	10.0 (9.2–10.9)
Gravidity			
1st Pregnancy (%)	33.3	35.7	31.3
2nd Pregnancy (%)	30.3	30.3	32.0
3rd Pregnancy or more (%)	36.3	34.0	36.7
Gestational age^b^			
2nd Trimester (%)	50.0	50.0	43.7
3rd Trimester (%)	50.0	50.0	56.3
Bed net used before study entry (%)	30.7	28.7	27.3
ITN used before study entry^c^ (%)	25.3	21.0	22.7
IPT use (before day 0) (%)	9.7	7.0	11.7

^a^At least 3 or more of the following symptoms: fever in the past 24 h, weakness/fatigue; muscle and/or joint aches, headache, convulsion, with parasitaemia of any density

^b^2nd Trimester were patients ≤24 weeks gestation and 3rd trimester >24 weeks gestation

^c^Women were provided with ITN at study start

The gametocyte prevalence at baseline was low, 2.3% (95% CI 1.1–4.7) in AL, 1.3% (95% CI 0.5–3.4) in DHAPQ and 0% (95% CI 0.0–1.3) in MQAS. The range of the gametocyte density, if present, was 40–440 gametocytes/µL. Gametocyte carriage remained low during the follow-up period and only appeared in AL (4/297; 1.3%) and DHAPQ (6/292; 2.1%) arms and none in MQAS. Use of preventive measures, i.e. ITN and IPTp, at recruitment was low (Table 1).

**Fig. 2:**
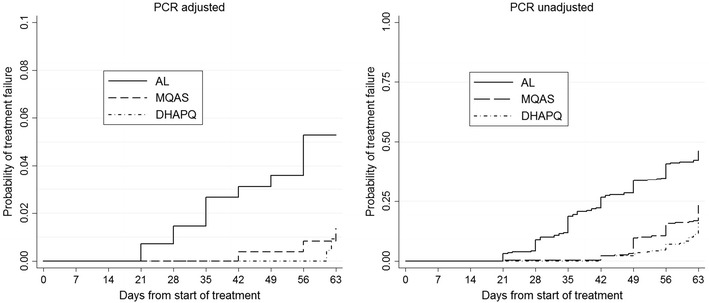
Time to PCR adjusted and unadjusted treatment failure in Zambian leg of PREGACT study in Nchelenge, Zambia (2010–2014)

**Fig. 3:**
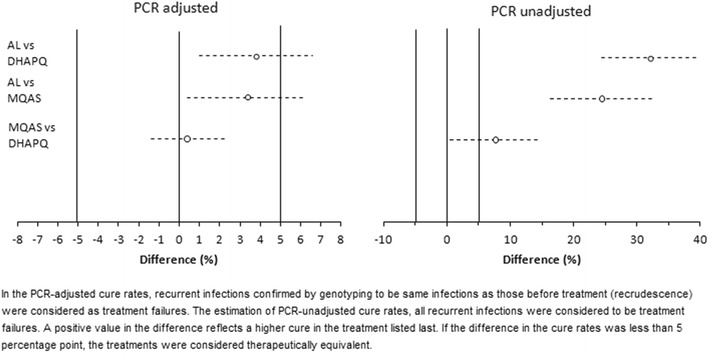
Pair-wise comparisons for PCR adjusted and unadjusted ACPR at days 63 (PP population). Nchelenge, Zambia (2010–2014) (95% CI)

In the PP analysis, the graphs for the global proportional hazards (PH) assumptions testing for treatment failure adjusted for several variables (treatment, anaemia, gestational age, gravidity, parasite density, maternal age and malaria symptoms at baseline) were roughly parallel and met the PH assumptions. The day 63 PCR-adjusted treatment failure rate was 4.7% (12/258) (95% CI 2.7–8.0) for AL, 1.3% (3/235) (95% CI 0.4–3.7) for MQAS and 0.8% (2/236) (95% CI 0.2–3.0) for DHAPQ (Table 2), with significant risk difference between AL and DHAPQ (p = 0.01) and between AL and MQAS (p = 0.03) treatments. Figure 2 which shows the time to PCR adjusted and unadjusted treatment failure confirms this difference. Figure 3 presents the risk difference computed for the pairwise comparisons conducted for PCR-adjusted and unadjusted treatment success rates at day 63. AL showed somewhat higher (about 3%) PCR-adjusted treatment failures. Therapeutic equivalence could be shown for MQAS and DHAPQ but not for AL as compared to the other 2 treatments. The ITT analysis gave similar results (Table 2). When considering recrudescence, i.e. treatment failure due to the reappearance of the same strain as identified by PCR analysis, its hazard was higher in patients treated with AL than in those treated with DHAPQ (HR: 10.47; 95% CI 2.18–50.19; p < 0.01) although the estimates were unstable probably due to the small or low number of observations. The hazard was not significantly different in the MQAS than in the DHAPQ arm (HR: 1.56; 95% CI 0.26–9.38; p = 0.63) (Table 3). The hazard of treatment failure was higher in younger women than in those over 20 years (HR: 5.07; 95% CI 1.01–25.43; p = 0.05). Higher parasite density at baseline was associated with a higher hazard of PCR-adjusted treatment failure (HR: 3.35; 95% CI 1.07–10.45; p = 0.04) (Table 3).

**Table 2:** Malaria treatment outcome of pregnant women with malaria episode in Nchelenge, Zambia (2010–2014)

	AL (N = 300)	MQAS (N = 300)	DHAPQ (N = 300)
*Efficacy outcomes, n (%)*			
Early treatment failure^a^	0	0	1
Late clinical and parasitological treatment failure^a^	126	60	36
Recrudescence	12	5	2
New infection	114	55	34
Adequate clinical and parasitological response^a^	132	175	199
Cannot be determined	42	65	64
Did not meet inclusion/exclusion criteria/missing	8	17	8
Treatment violations	1	0	1
No PCR sample	0	3	4
LTFU/died/withdrawn	33	45	51
*Treatment failure rate % (95% CI)*			
PP-analysis: PCR-unadjusted	48.4 (42.4–54.5)	23.9 (19.0–29.8)	16.3 (12.1–21.4)
PP-analysis: PCR-adjusted	4.7 (2.7–8.0)	1.3 (0.4–3.7)	0.8 (0.2–3.0)
ITT-analysis: PCR-unadjusted	47.6 (41.7–53.5)	25.1 (20.2–30.8)	16.5 (12.4–21.6)
ITT-analysis: PCR-adjusted	4.9 (2.9–8.2)	2.0 (0.9–4.6)	1.2 (0.4–3.5)
Placental malaria	N = 235	N = 228	N = 227
Acute infection, n (%)	3 (1.3)	2 (0.9)	0 (0.0)
Chronic infection, n (%)	75 (31.9)	70 (30.7)	67 (29.5)
Past infection, n (%)	148 (63.0)	139 (61.0)	146 (64.3)
No infection, n (%)	9 (3.8)	17 (7.5)	14 (6.2)

*LTFU* Lost to follow-up; *PP* per-protocol; *ITT* intention-to-treat

^a^Early Treatment Failure (ETF) defined as one of the following: (i) development of danger signs or severe malaria or worsening of clinical conditions on day 0, day 1, day 2 or day 3, in the presence of parasitaemia, (ii) parasitaemia on day 3 ≥count on day 0, (iii) parasitaemia on day 3 and fever (axillary temperature ≥37.5 °C). Late clinical failure (LCF) defined as (i) development of danger signs or severe malaria or worsening of clinical conditions on any day after day 3 in the presence of parasitaemia, without previously meeting any of the criteria of Early Treatment Failure or (i) presence of parasitaemia and fever on any day after day 3, without having previously meet the criteria of ETF. Late parasitological failure (LCF) defined as presence of parasitaemia on any day from day 4 onwards and axillary temperature <37.5 °C, without previously meeting any of the criteria of ETF or LCF. Adequate clinical and parasitological response (ACPR) defined as absence of parasitaemia at the end of the follow up period (day 63), irrespective of axillary temperature without previously meeting any of the criteria of early and late treatment failure. In the PCR-adjusted estimates, patients with late asexual parasite reappearance (with or without fever) are considered ACPR if the PCR analysis shows a new infection rather than a recrudescence. Placental malaria classified as: acute infection (parasite present, malaria pigment absent); chronic infection (parasites and malaria pigment present); past infection (no parasite but pigment present); no infection (both parasites and malaria pigment absent)

**Table 3:** Risk factors associated with recrudescence and new infection after anti-malarial treatment in pregnant women in Nchelenge, Zambia (2010–2014)

	Hazard ratio	95% CI	*p* value
*Risk factors associated with recrudescence*			
Treatment			
AL	10.47	2.18–50.19	
MQAS	1.56	0.26–9.38	
DHAPQ	1	1 (reference)	<0.01^a^
Maternal age (15–19 years)	5.07	1.01–25.43	0.05
Gestational age			
2nd Trimester	2.35	0.76–7.40	
3rd Trimester	1	1 (reference)	0.14
Gravidity			
Primigravidae	1.44	0.51–4.38	
Multigravidae	1	1 (reference)	0.47
Parasite density (>2000/µL)	3.35	1.03–10.10	0.04
Study treatment dosage (mg/kg for 3 days)	2.10	0.75–5.89	0.16
*Risk factors associated with malaria new infection*			
Treatment			
AL	4.71	3.10–7.15	
MQAS	1.59	1.02–2.46	
DHAPQ	1	1 (reference)	<0.01^a^
Maternal age (15–19 years)	1.78	1.26–2.52	<0.01^a^
Parasite density (>2000/µL)	1.46	1.09–1.94	0.01
Anaemia (Hb <11.0 g/dL)	1.56	1.05–2.32	0.03
Gravidity			
Primigravidae	1.11	0.80–1.54	
Multigravidae	1	1 (reference)	0.52
Study treatment dosage (mg/kg for 3 days)	1.00	0.74–1.37	0.98

^a^p value of the joint effect of treatment

New infections were more frequent in the AL (HR: 4.71; 95% CI 3.10–7.15; p < 0.01) and MQAS (HR: 1.59; 95% CI 1.02–2.46; p = 0.04) arms compared to the DHAPQ arm. The risk of re-infections was higher in women between 15 and 20 years (HR: 1.78; 95% CI 1.26–2.52; p < 0.01) than in women older than 20 years. Anaemic mothers had a higher hazard of new infection during follow up (HR: 1.56; 95% CI 1.05–2.32; p = 0.03). Similarly, mothers with higher parasite density a higher hazard of new infection (HR: 1.46; 95% CI 1.09–1.94; p = 0.01). All the other risk factors analysed were not significantly associated with new infection (Table 3).

Placental malaria infection (acute and chronic) was similar between the treatment arms (p = 0.47) (Table 2). Treatment allocation, AL and MQAS (DHAPQ as reference group), was not significantly related to placental malaria (OR 1.22; 95% CI 0.45–3.31 and OR 0.76; 95% CI 0.34–1.72, respectively) (p = 0.58). Women with recrudescence and new infections were at higher risk of placental malaria (OR 4.46; 95% CI 1.01–19.70; p = 0.05). Placental malaria was significantly higher in women between 15 and 20 years (OR 4.56; 95% CI 1.48–14.04; p = 0.01) than in women older than 25 years. Placental malaria was significantly associated with low birth weight (<2500 g) (OR 4.37; 95% CI 1.04–18.39; p = 0.04) but not with adverse birth outcomes (stillbirth, preterm, miscarriage, intrauterine fetal death). (OR 5.47; 95% CI 0.33–90.62; p = 0.24).

The study drugs were generally safe with a total of 7 SAEs for mother. A woman treated with MQAS died 41 days after treatment, probably because of meningitis. There were three SAEs in DHAPQ [low haemoglobin, measles and sickle cell mother in haemolytic crisis (vaso-occlusive)]. An additional SAE in the MQAS arm, severe vomiting, was considered related to study treatment and recovered completely. The other two in MQAS were asthmatic attack and pneumonia. They all recovered.

**Fig. 4:**
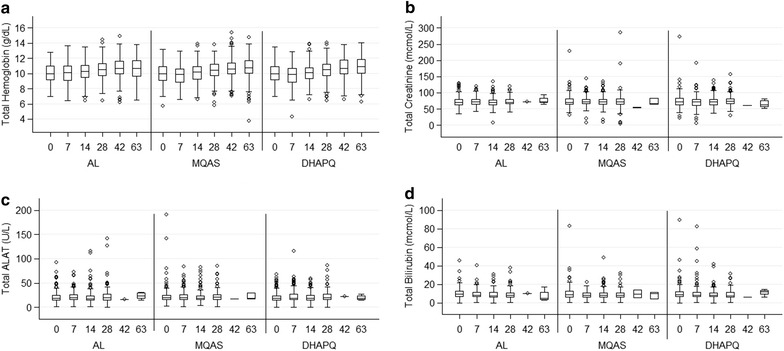
Box plots showing median and interquartile range as well as outlying values for total hematological and biochemical parameter levels in the *y*-axis and *x*-axis for each follow-up time point (Day 0 to Day 63) per treatment group (AL, MQAS, DHAPQ). **a** Comparison of hemoglobin level between treatment groups by day of follow-up. **b** Comparison of creatinine level between treatment groups by day of follow-up. **c** Comparison of ALAT level between treatment groups by day of follow-up. **d** Comparison of bilirubin level between treatment groups by day of follow-up

The proportion of women with AEs in each treatment arm (82.7% in AL, 84.9% in MQAS and 79.3% in DHAPQ) were not significantly different (p = 0.19) (Table 4). The drug-related AEs (dizziness, nausea, headache, vomiting and asthenia) were more common in the MQAS arm (67.9%; 95% CI 62.4–72.9) than the AL (12.7%; 95% CI 9.3–16.9) and DHAPQ arms (23.3%; 95% CI 18.9–23.4) (p < 0.01). There were significant differences in median Hb at day 7 between AL (10.1 g/dL) versus MQAS (9.9 g/dL)), p = 0.01; and AL vs DHAPQ (9.9 g/dL), p = 0.04; and at day 63 between AL (10.7 g/dL) vs DHAPQ (11.0 g/dL), p = 0.01 (Fig. 4). There were no significant differences in systolic and diastolic blood pressures between the treatment arms (p = 0.07 and p = 0.20 respectively). The median biochemical (creatinine, ALAT and bilirubin) safety values between treatment groups did not differ significantly during the follow-up period (p = 0.69, p = 0.92 and p = 0.88 respectively) (Fig. 4).

**Table 4:** Pregnant women with an adverse event till 63 days having received at least one malaria treatment dose in Nchelenge, Zambia (2010–2014)

Safety population	AL (N = 300)	MQAS (N = 299)	DHAPQ (N = 300)
At least one AE, n (%)	248 (82.7)	254(84.9)	238 (79.3)
Most common AEs^a^, n (%)			
Headache	136 (45.3)	142 (47.5)	134 (44.7)
Nausea	8 (2.7)	39 (13.0)	23 (7.7)
Cough	99 (33.0)	116 (38.8)	120 (40.0)
Asthenia	36 (12.0)	69 (23.1)	49 (16.3)
Dizziness	11 (3.7)	88 (29.4)	17 (5.7)
Vomiting	14 (4.7)	47 (15.7)	26 (8.7)
Abdominal pain	73 (24.3)	71 (23.7)	70 (23.3)
Musculoskeletal pain	43 (14.3)	47 (15.7)	30 (10.0)
Backache	51 (17.0)	40 (13.4)	28 (9.3)
Influenza	24 (8.0)	32 (10.7)	40 (13.3)
At least one related AE, n (%)	54 (18.0)	127 (42.5)	72 (24.0)
Most common related AEs^a^, n (%)			
Dizziness	5 (1.7)	72 (24.1)	8 (2.7)
Nausea	5 (1.7)	34 (11.4)	16 (5.3)
Vomiting	5 (1.7)	43 (14.4)	17 (5.7)
Asthenia	9 (3.0)	38 (12.7)	14 (4.7)
Headache	14 (4.7)	16 (5.4)	15 (5.0)
SAE n (%)	0	4 (1.3)	3 (1.0)
Related SAE, n (%)	0	1 (0.3)	0
At least one SAE which caused death, n (%)	0	1 (0.3)	0
Birth outcomes, n (%)			
Still birth	8 (2.8)	3 (1.1)	10 (3.7)
Miscarriage	0 (0)	2 (0.7)	1 (0.4)
Prematurity	13 (4.6)	6 (2.2)	10 (3.7)
Congenital abnormality	7 (2.6)	4 (1.5)	4 (1.6)

*AE* adverse event; *SAE* serious adverse event; *Related SAE* serious adverse event which the investigator classified as possibly, probably or definitely related to study drug

^a^AEs and related AEs recorded in, respectively, at least 10 and 5% of patients in any treatment group

There were 21 stillbirths: 8 (2.7%) in AL, 3 (1.0%) in MQAS and 10 (3.3%) in the DHAPQ arms and three miscarriages (two in MQAS, one in DHAPQ arms, and none in the AL arm). The preterm delivery was 4.3% in AL, 2.0% in MQAS and 3.3% in the DHAPQ arm. There were 15 congenital malformations [3 cleft lip and palate, one club foot, one ear tag, 6 polydactyl, one syndactyl, one umbilical hernia, one depression on parietal bone, one tongue tie) observed (4 (1.3%) in each of DHAPQ and MQAS arms and 7 (2.3%) in AL arm] with no significant difference between the arms (p = 0.54).

## Discussion

With the range of 0.8–4.7% recrudescences, the three artemisinin-based combinations used for the treatment of uncomplicated malaria in the second and third trimester of pregnancy were efficacious, in an area of high endemicity in Nchelenge district, Zambia. Therapeutic equivalence could be shown for MQAS and DHAPQ but not for AL as compared to the other two treatments. In Nchelenge Zambia, there were significantly more treatment failures in the AL arm compared to the other two arms, though AL efficacy was still above the 90% cure threshold recommended by WHO for adopting new anti-malarial treatments as policy [21]. In Uganda, in an area with malaria transmission as high as that in Nchelenge, AL administered to pregnant women was also extremely efficacious, with even less treatment failures (0.7%) than in this trial [22].

In Zambia, ACT has been shown to have excellent cure rates among children and adults [23, 24]. Their efficacy, determined by the drug partnering an artemisinin derivative, namely mefloquine, lumefantrine, and piperaquine for the treatment tested in this study, usually exceeds 95% [25]. However, there have been reports pointing to the effect of the physiological changes during pregnancy, e.g. as increased volume of distribution, reduced gut motility, possibly altering drug disposition and metabolism, and thus leading to incorrect dosing [26–28]. This does not seem to apply to the results observed in Nchelenge as treatment efficacy was very high, possibly due to the underlying anti-malarial immunity in the Nchelenge district population, including pregnant women, due to the intense malaria transmission and high exposure to infection. The importance of pre-existing immunity on the therapeutic response is also supported by the association between treatment failure (both new infections and recrudescences) and young age [29]. Also transmission intensity may not influence the risk difference between treatments but may influence individual failure rates.

Pregnant women have an increased susceptibility to malaria, and this susceptibility is greatest in the first pregnancy (primigravidae) [30]. The decreasing prevalence and intensity of infection in successive pregnancies mirrors the acquisition of antibody immunity to the variant surface antigens, expressed on the parasitized red blood cells infecting the placenta. Antibodies titres against VSA-PAM are associated to clinical outcomes [31, 32] and opsonizing antibodies that allow phagocytic clearance of infected erythrocytes are associated with a better treatment outcome in pregnant women [33]. Results from Nchelenge and other studies suggest that antibodies to VSA-PAM might have important roles in determining both pregnancy outcomes and the effectiveness of anti-malarial drugs in pregnancy. Other factors such as cellular immunity, cytokines, and hormonal changes might also influence outcomes in pregnancy [29] and also affect treatment outcome.

In Nchelenge, pregnant women treated with AL had a higher risk of new infection than the other two treatments. This is probably due to the shorter post-treatment prophylaxis offered by lumefantrine which is eliminated more rapidly [34] than piperaquine [35]. When the artemisinin component is rapidly eliminated, a new infection would encounter only the partner drug and this may explain the association between the risk of new infection and treatment given. It indirectly confirms that the distinction between recrudescence and new infection and genotyping is reasonably reliable. Considering that Nchelenge women who experienced a new infection during follow up had a higher risk of acute or chronic placenta malaria, both conditions associated to the delivery of low birth weight babies, a longer post-treatment prophylaxis would be extremely important in this area of intense malaria transmission. Therefore, DHAPQ could be preferentially chosen for such conditions, while AL could be used where transmission is low.

Recrudescence may easily occur in the context of emergence or spread of parasite resistance to a given anti-malarial when the partially efficacious anti-malarial may fail to clear the resistant strain or simply select for mutant parasites. In Zambia, artemisinin resistance has not been reported yet. Recrudescence can be caused by the parasites surviving the effect of a shorter-acting ACT [6], in this case AL. Low study drug dosage may play an important role in recrudescence in the AL group as the point estimate indicates low study drug dosage suggests a double independent risk for recrudescence. However, the power of the study was to assume a clear association. Besides parasite sensitivity to drug and the level of the concentration of the drug in the blood, host immunity and parasite density at presentation contributes to positive treatment outcome. Immunity can be affected by different factors, including age, body temperature, pregnancy and parity [29, 36]. The Nchelenge study has shown that younger women and high malaria parasite density at baseline are associated with recrudescence and could not demonstrate a significant association between treatment failure and parity.

Other studies have shown that high parasite density at presentation is associated with treatment failure [30–33, 36–38] and that age, temperature and parasite density are predictors of anti-malarial treatment failure [36].

The three artemisinin-based combinations tested are generally safe in second and third trimester of pregnancy in Zambia. Patients on MQAS had higher rates of treatment-related AE. Dizziness was the most common, followed by vomiting and weakness. Dizziness has been reported even in other studies as related to MQ treatment [39]. On the pregnancy outcomes, there was no significant difference between treatments for stillbirths, miscarriages, congenital malformations and prematurity, a finding similar to those of other studies on AL [22, 40, 41], mefloquine [5] and DHAPQ [42].

This trial was done in an area where the majority of the population practice farming and fishing as a source of livelihood and they migrate to farming areas for a considerable period [43], possibly explaining the relatively high number of lost-to-follow-ups and withdrawals. Nevertheless, considering that the post-treatment follow up was up to day 63 and that pregnant women are a group particularly difficult to follow, the sample size had been estimated assuming a dropout rate of 20%, while the actual figure was 16%. Such a relatively high dropout rate is unlikely to have had a major influence on the trial’s results as the patients excluded and those included did not differ significantly on their baseline characteristics.

## Conclusions

The study has shown that both AL and DHAPQ were well tolerated in second and third trimester pregnant women, with low treatment failures. MQAS was less well tolerated than the other two treatments though it had similar low treatment failure. DHAPQ seems to be well tolerated and has low treatment failure with a longer post-treatment prophylaxis. As new infections can be prevented by a long acting partner drug to the artemisinins, DHAPQ should be preferred where transmission is intense as in Nchelenge while and in areas of low transmission intensity AL or MQAS may be used.

## Abbreviations

ACPR: adequate clinical and parasitological response
AE: adverse events
AL: artemether–lumefantrine
ACT: artemisinin-based combination treatment
DHAPQ: dihydroartemisinin–piperaquine
ETF: Early Treatment Failure
e-CRF: electronic clinical record form
Hb: haemoglobin
HR: hazard ratio
ITT: intention-to-treat
IQR: interquartile range
ITN: insecticide treated net
LTFU: lost to follow-up
MQAS: mefloquine–artesunate
OR: odds ratio
PP: per-protocol
PCR: polymerase chain reaction
SAE: serious adverse event
